# The psychometric properties of a new oral health illness perception measure for adults aged 62 years and older

**DOI:** 10.1371/journal.pone.0214082

**Published:** 2019-04-10

**Authors:** Suchitra Nelson, Jeffrey M. Albert, Yiying Liu, David Selvaraj, Shelley Curtan, Kelli Ryan, Andres Pinto, Farida Ejaz, Peter Milgrom, Christine Riedy

**Affiliations:** 1 Case Western Reserve University School of Dental Medicine, Cleveland, Ohio, United States of America; 2 Case Western Reserve University School of Medicine, Cleveland, Ohio, United States of America; 3 Benjamin Rose Institute on Aging, Cleveland, Ohio, United States of America; 4 University of Washington School of Dentistry, Seattle, Washington, United States of America; 5 Harvard School of Dental Medicine, Boston, Massachusetts, United States of America; Centre Hospitalier Regional Universitaire de Tours, FRANCE

## Abstract

**Background:**

Based on the Common-Sense Model of Self-Regulation (CSM), a new integrated Illness Perception Questionnaire Revised for Dental Use in Older/Elder Adults (IPQ-RDE) was developed for single and multiple dental conditions. This study describes psychometric properties of the IPQ-RDE for adults 62 years and older.

**Methods:**

Participants (n = 198) living in 16 subsidized housing facilities completed the IPQ-RDE and a questionnaire assessing their socio-demographics, frequency of dental visits, perceived condition of teeth/gums, depression, social support, and oral health quality of life (OHQOL). Participants received dental screening for presence/absence of teeth, coronal and root caries, and periodontitis. The 43-item IPQ-RDE was tested for internal (construct, discriminant) and external validity (concurrent, construct, discriminant, predictive) and reliability (internal consistency).

**Results:**

Confirmatory factor analysis demonstrated that a ten-factor model in accordance with the CSM framework (identity, consequences, control, timeline, illness coherence, treatment burden, prioritization, causal relationship, activity restriction, emotional representations) had good construct validity based on significant factor loadings and acceptable model fit (RMSEA = 0.065, CFI = 0.902). Edentulous participants had significantly higher mean factor scores (inaccurate perception) for overall IPQ-RDE and four constructs indicating concurrent validity. Discriminant validity was suggested by non-relationship with external measures (education, dental visit frequency). Predictive validity was indicated by the negative correlation of most constructs with OHQOL suggesting that inaccurate perception was related to lower quality of life. Internal consistency of eight IPQ-RDE constructs was excellent (Cronbach’s alpha > 0.73).

**Conclusions:**

The IPQ-RDE is a valid and reliable new measure for assessing older adult’s perception of dental conditions. It can be an important tool for oral health behavioral research to restructure older adult’s perception of dental conditions, and subsequently prevent tooth loss and improve oral health quality of life.

## Introduction

The Common Sense Model of Self-Regulation (CSM) [1] has been shown to be useful in conceptualizing the self-management of chronic conditions. The model posits that individuals form a cognitive and emotional representation (perception) based on abstract and concrete sources of information available to them in order to self-manage their illness. Thus, in response to an illness threat, the individual’s perception will guide a coping response and action planning [1]. The Revised Illness Perception Questionnaire (IPQ-R) has been used to measure illness representations among patients with a single disease [2]. Recently, research suggests a questionnaire incorporating multiple diseases is essential for older adults [3, 4]. Management of multiple medical conditions (or similarly multiple oral diseases) is considered a critical challenge for future health care delivery [5, 6]. A qualitative study suggested that having multiple medical conditions influences the IPQ-R domains of cognitive representation [3]. Patients had difficulty linking individual symptoms to a particular disease, which impacted representations of identity and cause, and in turn reduced illness coherence. The study identified potential new domains of illness perception, i.e. perceived priorities among conditions or burden from multiple conditions that could influence treatment related decision-making and management strategies. To measure the perceived impact of multiple medical diseases, the Multimorbidity Illness Perceptions Scale (MULTIPleS) was developed [7] which has five domains (treatment burden, prioritizing conditions, causal links, activity limitations, and emotional representation). Although the instrument demonstrated acceptable test-retest reliability, further research was warranted for construct and predictive validity [7].

Oral health problems are common [8–10] and cumulative throughout life; the disease burden increases as one gets older[11]. The progression of oral diseases can adversely affect systemic health [12]. Despite profound impact of oral health status on quality of life [12], many older adults accept their oral health problems as an inevitable process of aging and do not seek dental care [13, 14]. Nevertheless, few oral health self-reported measures exist for older adults, with the existing measures focusing on oral health related quality of life [15], and factual knowledge, attitudes, self-efficacy about oral health [16, 17]. However, these measures are mainly assessing the consequences of dental conditions rather than the older adult’s perception and understanding of the chronicity of their underlying oral disease. Previously, we developed and tested a revised illness perception questionnaire for dental (IPQ-RD) to assess parental perception of children’s dental caries [18]. We extend this work to older adults using the IPQ-R and MULTIPLeS each with unique domains that can be used for individuals with single or multiple diseases. The objective was to develop and psychometrically test an integrated illness perception measure for oral conditions to increase its broad applicability to an older adult population.

## Methods

Based on the CSM framework, a new integrated Illness Perception Questionnaire Revised for Dental Use in Older Adults (IPQ-RDE) was developed and tested in two phases: **Phase I** was to develop the questionnaire with content experts and cognitive interviews with participants; **Phase II** was to conduct psychometric testing of the final questionnaire in a larger sample of participants.

### Phase I: Development of the illness perception questionnaire revised for dental use in older adults (IPQ-RDE)

The Patient-Reported Outcome Measurement Information System (PROMIS) Instrument Development and Validation Standards [19] served as a guideline for the psychometric assessment. Building on our previous experience [18], the team developed an initial draft of an illness perception instrument in English for use with older adults with single and multiple dental conditions. Content experts (dentist, psychologist, social scientist/gerontologist, epidemiologist) integrated the IPQ-R and MULTIPleS and revised them as follows: 1) combined items from the two questionnaires to be appropriate for oral diseases (e.g., “my illness” replaced with “my tooth/mouth condition”); 2) added items unique for oral diseases (e.g., “my tooth/mouth condition is as serious as my medical condition”); 3) modified items focusing on reading level and clarity (e.g., “having financial consequences” modified to “causing money problems”); and 4) since both IPQ-R and MULTIPleS had emotional representation they were merged into one domain. The initial draft of the IPQ-RDE contained 13 domains (51 items) based on the IPQ-R and MULTIPLeS measures.

This initial draft of IPQ-RDE was pretested using cognitive interviewing with "think-aloud" and "verbal probing" techniques to identify item wording, question order, visual design, and navigation problems to ensure content validity [20]. The first round included cognitive interviews with 12 older adults recruited from two randomly selected independent housing facilities. One-on-one interviews (≈60 minutes) were conducted at the site by study staff trained in cognitive interviewing. Participants were probed for comprehension of items and understanding of recall and response options. The staff audio recorded the session, which were then transcribed and reviewed by two study staff who focused on the results of the comprehension and probing to refine each item in the IPQ-RDE. Additionally, data on the interview length and interviewer feedback regarding respondent burden was used to refine the questionnaire. This round of interview indicated that the majority of participants preferred “my oral disease” rather than “my tooth/mouth condition” and that some questionnaire items were redundant. The content experts revised the questionnaire taking into account participants input and examining item wording that resulted in a 43-item IPQ-RDE. A second round of cognitive interviews with another 6 older adults from the same housing facilities was conducted. Participants indicated that “my oral disease” should be changed to “my oral health condition” and the questionnaire length as being appropriate. Subsequently, the 43-item questionnaire was finally revised (with change to “my oral health condition”) for the psychometric testing (See S1 Text for final IPQ-RDE questionnaire).

#### Specifics of the final 43-item illness perception questionnaire revised for dental use in older adults (IPQ-RDE)

Because older adults are likely to have multiple oral and systemic diseases, the IPQ-RDE is an integrated measure based on the IPQ-R [2] and the MULTIPleS [7] measures. The IPQ-RDE include domains of cognitive (12 constructs) and emotional representation (1 construct) for a total of 13 constructs. The cognitive constructs of the IPQ-RDE from the original IPQ-R and MULTIPleS framework included the following: *Identity*, labeling of oral conditions and its symptoms; *Timeline (Chronic and Cyclical)*, beliefs about oral conditions being acute, chronic, or cyclical in nature; *Consequences*, beliefs about the impact of oral conditions physically and socially; *Control (Personal and Treatment)*, beliefs about whether oral conditions can be cured or kept under control; *Illness Coherence*, whether the individual has a clear understanding of oral conditions and symptoms associated with it; *Treatment Burden*, beliefs about the effectiveness of treatment for one or more oral health conditions and the burden associated with the treatments; *Prioritization*, beliefs about prioritizing more than one oral health condition; *Causal Relationship*, beliefs about the relationship between multiple oral conditions and links to medical conditions; *Activity Restriction*, beliefs about time spent and effects on restricting other daily and social activities; *Cause*, perception of the underlying cause of oral conditions; and the single construct *Emotional Representation*, the individuals’ emotional response (e.g. worry, anger) to their single or multiple oral conditions. Each item was scored on a 5-point Likert scale (strongly agree to strongly disagree) with a poorer (inaccurate) perception represented by a higher score. The *cause* construct was not included in the psychometric analyses because as reported in previous literature it is considered to have its own factor structure and is excluded [21] or analyzed apart from the other constructs [22]. Thus, the psychometric analysis included 12 constructs with 43 items.

### Phase II: Study design and participants

The 43-item IPQ-RDE questionnaire was tested in a separate sample of participants from sixteen independent housing facilities in Northeast Ohio between January 2016 and April 2017. All 16 facilities provide low-income, older (62 and older) tenants with subsidized housing funded by U.S. Housing and Urban Development (HUD, Section 202), and other low-income housing tax credits. Eight of the 16 facilities also included private pay (non-HUD) market rate rental apartments for tenants above HUD’s income eligibility guidelines. Service coordinators at each of the housing facility introduced the dental study to their tenants through flyers, newsletters, and at tenant meetings. Participants volunteered and signed up at these events to be contacted by the research staff, who then obtained informed consent and set up dental examinations and interview appointments. Questionnaires were administered to the participant by trained study staff. The study was approved by the University Hospitals Cleveland Medical Center Institutional Review Board and informed consent was obtained from participants. This manuscript conforms to STROBE guidelines for observational studies.

#### Variables/measures collected for psychometric analyses

Participants completed the following: (1) Illness perception questionnaire revised for dental use in older adults (IPQ-RDE); clinical exam where the participant received a dental screening from a licensed dentist; and a participant questionnaire. The *IPQ-RDE* was administered by the study staff. *Dental screening exam* included assessments for untreated coronal and root caries: decayed teeth ≥ 1 yes, 0 no); clinical attachment loss (CAL) and pocket depth were used to assess the severity of periodontitis (none/mild, moderate, severe) using previous definition [23]. *Socio-demographics* included *age* in years, *race* (Black, non-black), *marital status* (single/widowed, married), *level of education* (≤ high school diploma/GED vs. > high school diploma/GED), and *housing status* (HUD vs. non-HUD). *Psychosocial variables* were measured on a three- or five-point Likert type scale: social support, a 7-item score [24]; and PROMIS short form depression and anxiety measure for adults, an 8-item score [25], with higher scores indicating greater social support or depression. The *Geriatric Oral Health Quality of Life*, a 12-item score, assessed on a three-point Likert scale was also used with a higher scores indicating better quality of life [15]. The frequency of dental visits (often: ≤ 1 year vs. none/rare > 1 year) and perceived condition of teeth and gums (good/excellent vs. fair/poor) were self-reported by participants.

#### Psychometric analysis

Only 12 IPQ-RDE constructs (43 items) were included for the psychometric testing. Sample size estimates were based on the subjects-to-variables ratio of no lower than 5 [26], and our participant sample size met this criteria. Statistical significance was assessed at the .05 alpha level.

**Fig 1:**
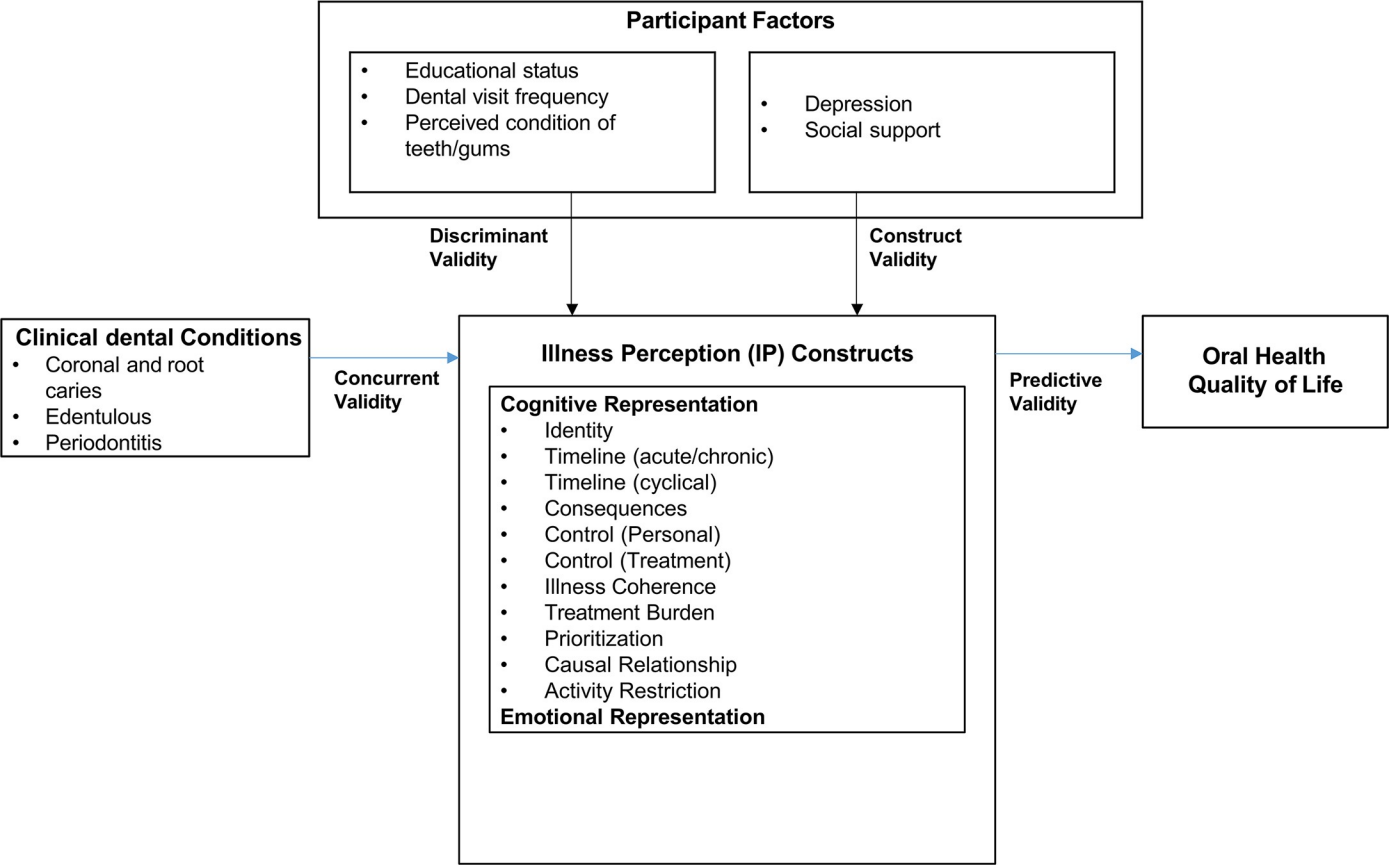
Model for psychometric testing.

Fig 1 outlines the process of the IPQ-RDE testing and summarized as follows: (1) internal validity: evaluation of the instrument’s hypothesized factor structure and assessment of the model’s fit to the data, as well as discriminant validity (i.e. inter-correlations between the IPQ-RDE constructs) based on the constructs of the CSM framework; (2) external validity: evaluation of the IPQ-RDE with other measures. *Concurrent validity* was examined using the clinical data (coronal and root caries, edentulous, periodontitis) from the dental screenings. *Construct validity* was examined using several standardized measures (i.e. social support, depression) that can validate some of the IPQ-RDE constructs, e.g. depression with emotional representation. *Discriminant validity* was examined using sociodemographic and oral health characteristics (education, dental visit frequency) and participant-reported condition of tooth and gums. It was hypothesized that older adults with caries, were edentulous, high school education or less, fewer dental visits, and poor condition of tooth and gums will have inaccurate illness perception compared to their counterparts. Finally, we examined the *predictive validity* of the IPQ-RDE with oral health quality of life. It was hypothesized that older adults with poorer (inaccurate) illness perception will have a reduced oral health quality of life; and (3) internal consistency (reliability).

Confirmatory factor analysis (CFA) was conducted in MPlus (Version 6.12) to assess internal validity of the IPQ-RDE. Factor loadings were estimated using the weighted least squares with adjustments for the mean and variance (WLSMV) method. Internal validity is indicated by large values for factor loadings or high residual correlation with other items across constructs. Items with a standardized factor loading of less than 0.2 [22, 27], as a conservative criterion, and with substantial residual correlation (>0.2) with other items, were considered for removal. According to this criteria, in preliminary CFA analysis, three questionnaire items were removed: #5 “My oral health condition will improve over time” in Timeline, #18 “The symptoms of my oral disease are puzzling to me” in Illness Coherence, and #34 “An oral disease is a side effect of medication used for a medical condition” in Causal Relationship. Internal validity was also assessed through the following model fit criteria: root mean square error of approximation (RMSEA < 0.08 for acceptable fit) and comparative fit index (CFI > 0.90 for excellent fit) [28].

Additional validity tests were conducted based on the CFA. We used the simple and transparent assessments of the factor scores (in lieu of trying to fit more complex CFA models to conduct such tests): 1) Spearman correlations among factors (using standardized factor scores) were computed to assess discriminant validity; low correlations indicate appropriately distinct constructs though some constructs are recognized to be conceptually related; 2) Relationship between factor scores and other participant variables were assessed to test external validity: t-tests for mean difference in factor scores between levels of each binary variable (coronal/root decay, edentulous status, periodontitis, socio-demographics, dental visit frequency, perceived tooth/gum condition), Spearman’s correlation for psychosocial variables and oral health quality of life; and 3) Residual correlation matrices were obtained to assess the local independence assumption of the CFA model; a residual correlation of greater than 0.2 was flagged as indicating a plausible violation of this assumption. To test the assumption of unidimensionality for the CFA model, we conducted exploratory factor analyses to obtain the eigenvalues for each factor. A unidimensional proportion (the magnitude of the largest eigenvalue divided by the total of all the eigenvalues for the factor) of less than 0.2 indicates a possible departure from unidimensionality. Cronbach’s alpha assessed the internal consistency of each construct with a value ≥ 0.70 indicating reliability.

In addition, CFA models with observed grouping (categorical) covariates (equivalent to two-parameter item response theory (IRT) models, [29]) were fit. These models provide estimates of the intercept (or scale cutpoints) and slopes (or item loadings) for each item and for each covariate level. We used single-factor models to study group differences for each construct. The fitted IRT models and a constructed chi-square statistic were used to test differential item functioning (DIF); namely, that model (intercept and/or slope) parameters are different for different subpopulations (age, race, and housing group).

As an alternative supplementary approach, we conducted some of the above tests using the Rasch model. Specifically, we carried out DIF analyses under the Rasch model (with common versus separate parameters for each group) for the same baseline variables as above. We also computed item fit (likelihood ratio chi-square) statistics, and corresponding p-values, for each item for each IPQ-RDE construct (in this case, dichotomizing item scores as required by the approach used). Rasch analyses were carried out using the SAS IRT Procedure. As we consider the Rasch model to be overly restrictive for our data context [30] we did not base our conclusions on the Rasch model results; rather, the results are provided (in the S1 and S2 Tables) for reference for the interested reader.

## Results

Based on the CSM framework, the IPQ-RDE measure was developed and finalized in Phase I, and psychometrically tested in Phase II of the study. In Phase II, a total of 198 (HUD: 100; non-HUD: 98) participants completed the IPQ-RDE and the participant questionnaire. However, 7 participants with missing IPQ-RDE data were excluded, and psychometric testing was conducted on 191 participants with complete data. Table 1 shows that participants were predominantly female (75%), white (55%), single/widowed (93%), greater than high school education (52%). Dental screening indicated that 27% were edentulous, 58% and 42% with untreated coronal and root caries decay respectively, and 72% with moderate to severe periodontitis. Participant self-report indicated that 60% had rare dental visits, 62% and 43% reported that the condition of their teeth and gums were fair or poor respectively.

**Table 1:** Sociodemographic and clinical characteristics of Phase II participants (N = 198).

Sociodemographic characteristics	
**Gender (N = 198)**	
Female	148 (75%)
Male	50 (25%)
**Age (N = 194)**	
Years, Mean ± SD	72.14 ± 9.02
**Race/ethnicity (N = 194)**	
Black	87 (45%)
Non-Black	107 (55%)
**Marital status (N = 198)**	
Single/widowed	184 (93%)
Married	14 (7%)
**Level of education (N = 198)**	
≤ High school diploma / GED	96 (48%)
> High school diploma	102 (52%)
**Housing (N = 198)**	
HUD	100 (51%)
Non-HUD	98 (49%)
**Clinical characteristics**	
**Teeth group (198)**	
With Teeth	144 (73%)
No teeth (Edentulous)	54 (27%)
**Untreated coronal caries (N = 144)**	
Yes	84(58%)
No	60(42%)
**Untreated root caries (N = 144)**	
Yes	61(42%)
No	83(58%)
**Periodontitis (N = 139)**	
Severe	38 (27%)
Moderate	63 (45%)
None/Mild	38(27%)
**Patient-reported characteristics**	
**Dental visit frequency (N = 198)**	
Often(≤ 1 year)	80 (40%)
Rare(>1 year)	118 (60%)
**Perceived teeth condition (N = 143)**	
Good/excellent	54 (38%)
Poor/fair	89 (62%)
**Perceived gum condition (N = 198)**	
Good/excellent	112 (57%)
Poor/fair	86 (43%)

### Internal validity

The IPQ-RDE was tested for construct validity with the original 12 constructs using confirmatory factor analysis (CFA). During preliminary assessment to improve model fit similar constructs were combined (i.e. timeline chronic and timeline cyclical; control, personal and treatment) for a total of 10 constructs, and three redundant items were removed due to low factor loadings. In the final model, several correlation parameters were added to account for some moderate residual correlations with the goal of keeping residual correlations below 0.2 without undermining other aspects of the model fit. Table 2 indicates that all items had significantly high standardized factor loadings suggesting that the 10 factor model fit the data in an acceptable (RMSEA = 0.065) to excellent (CFI = 0.902) manner. Unidimensionality scores for all CFA factors were greater than 0.38 indicating that the set of items represented a single underlying IPQ-RDE construct.

**Table 2:** Confirmatory factor analysis of IPQ-RDE among older adults (10 factors).

Factors (illness perception constructs) & items	Standardized factor loading*	Standard error	Unidimensionality†
***Identity (2 items)***			0.84
1: My oral health condition is an illness with symptoms generally of an intense nature.	0.85	0.03	
2: My oral health condition is an illness with many symptoms.	0.89	0.02	
***Timeline (5 items)***			0.38
3: My oral health condition will last a short time.	0.25	0.08	
4: I expect to have an oral health condition for the rest of my life.	0.49	0.06	
21: The symptoms of my oral health condition may change from day to day.	0.74	0.04	
22: I cannot predict how my oral health condition will change over time.	0.51	0.06	
23: I go through cycles in which my oral health condition gets better and worse	0.59	0.05	
***Consequences (6 items)***			0.57
6: My oral health condition is a serious problem.	0.82	0.03	
7: My oral health condition is as serious as any other medical condition.	0.75	0.04	
8: My oral health condition has major consequences on my life such as chewing, speaking or aesthetic problems.	0.82	0.04	
9: My oral health condition has much effect on my daily life.	0.77	0.04	
10: My oral health condition has a big effect on how others think about me.	0.75	0.03	
11: My oral health condition has caused money problems for me or my family	0.58	0.05	
***Control (6 items)***			0.63
12: There is a lot I can do to control my symptoms.	0.75	0.03	
13: What I do decides if my oral health condition gets better or worse.	0.72	0.03	
14: I have the power to influence the outcome of my oral health condition.	0.70	0.04	
15: There is a lot that can be done to improve my oral health condition.	0.82	0.03	
16: My treatment will help make my oral health condition better.	0.93	0.02	
17: My treatment can control my oral health condition.	0.89	0.02	
***Illness Coherence (2 items)***			0.79
19: My oral health condition makes sense to me.	0.91	0.06	
20: I have a clear picture or understanding of my oral health condition.	0.66	0.05	
***Treatment Burden (5 items)***			0.47
24: I feel overwhelmed by the treatment for my oral health condition.	0.77	0.04	
25: It is difficult to visit a dentist for my oral health condition when I have a problem.	0.68	0.06	
26: Visiting a dentist for each of my oral health conditions would cause more problems.	0.60	0.05	
27: Having more than one oral health condition would make treatments less effective.	0.67	0.04	
28: Having more than one oral health condition would make it difficult to get the best available treatment.	0.61	0.06	
***Prioritization (3 items)***			0.67
29: With an oral health condition, one is more serious than the others.	0.69	0.05	
30: With an oral health condition, one takes over the others.	0.79	0.05	
31: With an oral health condition, one has more of an effect on my life than the others.	0.79	0.05	
**Causal Relationship (3 items)**			0.51
32: The causes of oral health conditions are linked.	0.76	0.05	
33: One oral health condition causes another.	0.65	0.05	
35: My oral health condition can be linked to a medical condition.	0.32	0.07	
**Activity Restriction (3 items)**			0.76
36: Time spent managing my oral health condition makes it difficult to do my daily activities.	0.83	0.03	
37: Time spent managing my oral health condition has limited my activities.	0.91	0.02	
38: Time spent managing my oral health condition has reduced my social life.	0.84	0.03	
**Emotional Representations (5 items)**			0.55
39: I get really sad and upset when I think about my oral health condition.	0.84	0.03	
40: My oral health condition makes me feel angry.	0.80	0.03	
41: My oral health condition worries me.	0.73	0.04	
42: Having more than one oral health condition makes someone more bad-tempered.	0.57	0.05	
43: When I feel sad or down, managing my oral health condition is hard to do.	0.69	0.04	

* All factor loadings were significant at the *α* = 0.05 level.

^†^ Unidimensionality is the magnitude of largest eigenvalue divided by the total of all the eigenvalues for the factor, and is interpreted as the proportion of total item variance explained by the single factor. A value > 0.2 is considered to support unidimensionality (adequacy of a single factor for the items)

The CFA model included correlations for item pairs (6, 20), (9, 27), (26, 35)

Table 3 indicates that some of the IPQ-RDE constructs were recognized to be conceptually related as shown by significant correlations (e.g. identity and consequences: r = 0.82). However, all IPQ-RDE constructs were empirically distinct based on the threshold of correlation coefficients < 0.85 [31] indicating discriminant validity.

**Table 3:** Spearman’s correlations between illness perception constructs (10 factor model) among older adults.

	Identity	Timeline	Conseq-	Control	Illness cohere-	Trtment burden	Prioritization	Cause relation	Act-restrict	Emo-repres-
**Identity**	1.00									
**Timeline**	-0.70*	1.00								
**Conseq-**	0.82*	-0.71*	1.00							
**Control**	0.34*	-0.52*	0.55*	1.00						
**Illness cohere-**	0.09	-0.17*	0.20*	0.66*	1.00					
**Trtment burden**	-0.62*	0.68*	-0.55*	-0.15*	0.06	1.00				
**Prioritization**	-0.47*	0.51*	-0.56*	-0.19*	-0.04	0.56*	1.00			
**Cause-relation**	0.37*	-0.53*	0.57*	0.77*	0.39*	-0.30*	-0.52*	1.00		
**Act-restrict**	-0.60*	0.43*	-0.51*	-0.07	0.13	0.72*	0.45*	-0.19*	1.00	
**Emo-repress-**	-0.41*	0.51*	-0.46*	-0.10	0.20*	0.77*	0.39*	-0.14*	0.83*	1.00

*Significant at *α* **= 0.05**

### External validity

Table 4 shows concurrent validity between the IPQ-RDE constructs and clinical measures (edentulous, untreated coronal/root decay). Comparison of factor scores using t-tests indicated that older adults who were edentulous (i.e. no teeth) had significantly inaccurate perception (i.e. higher factor scores) for consequences, control, causal relations, and overall IPQ-RDE versus those who had teeth. Interestingly, those without teeth had a significantly more accurate perception of timeline and prioritization (i.e. lower factor scores) compared to those with teeth. The mean IPQ-RDE individual and overall factor scores were not significantly different between older adults with and without untreated coronal/root decay. The only construct that was significant for periodontitis was identity with those having the oral disease having lower factor scores (indicating accurate perception) compared to those without the disease.

**Table 4:** Relationship between illness perception constructs and clinical variables among older adults.

Illness perception constructs (10 factor model)												
	Decay—Coronal			Decay—Root			Edentulous			Periodontitis		
	yes (mean)	no (mean)	P-value	yes (mean)	no (mean)	P- value	Yes (mean)	No (mean)	P- value	Yes (mean)	No (mean)	P- value
**n**	81	57		59	79		53	138		93	86	
**Identity**	-0.16	0.065	0.167	-0.16	0.004	0.311	0.22	-0.065	0.057	-0.12	0.17	0.036*
**Timeline & cycle**	0.11	0.12	0.904	0.12	0.11	0.987	-0.34	0.11	0.001*	0.11	-0.12	0.76
**Consequence**	-0.13	-0.071	0.703	-0.15	-0.076	0.651	0.35	-0.11	0.002*	-0.11	0.16	0.059
**Control personal & treatment**	-0.102	-0.20	0.549	-0.073	-0.19	0.447	0.49	-0.14	<0.001*	-0.045	0.093	0.34
**Illness coherence**	-0.001	-0.047	0.767	0.069	-0.086	0.322	0.17	-0.020	0.16	0.14	-0.091	0.078
**Treatment burden**	0.018	0.012	0.969	0.12	-0.061	0.279	-0.035	0.015	0.73	0.041	-0.033	0.591
**Prioritization**	0.097	0.10	0.979	0.11	0.088	0.868	-0.27	0.098	0.009*	0.12	-0.14	0.052
**Causal relation**	-0.14	-0.12	0.867	-0.17	-0.11	0.674	0.41	-0.13	<0.001*	-0.06	0.089	0.247
**Activity restrict**	0.049	-0.19	0.167	0.073	-0.14	0.212	0.14	-0.048	0.21	-0.052	0.029	0.568
**Emotional representation**	-0.001	-0.025	0.888	0.041	-0.050	0.590	0.038	-0.011	0.744	0.008	-0.008	0.909
**Overall Factor Score**	-0.026	-0.034	0.899	-0.001	-0.051	0.436	0.12	-0.030	0.009*	0.003	0.015	0.820

* Significant at *α* = 0.05 (t-test was used to test for mean differences)

Table 5 (discriminant validity) shows that older adults with high school education or less had significantly inaccurate perception (higher factor scores) only for causal relationship, while those with no/rare dental visits had significantly inaccurate perception only for activity restriction compared to their counterparts. Older adults with poor self-reported teeth/gum condition had significantly inaccurate perception for timeline, treatment burden, prioritization, activity restriction, and emotional representation compared to adults with good reported teeth/gum health.

**Table 5:** Relationship between illness perception constructs (10 factor model) and self-reported participant variables.

	Education			Dentist Visit -frequency			Perceived teeth health			Perceived gum health		
	>high school (mean)	≤high school (mean)	P- value	Often (mean)	Rare (mean)	P-value	Good (mean)	Poor (mean)	P-value	Good (mean)	Poor (mean)	P-value
**n**	99	92		77	114		53	84		108	83	
**Identity**	0.026	0.001	0.853	0.12	-0.061	0.174	0.41	-0.37	<0.001*	0.26	-0.30	<0.001*
**Timeline & cycle**	0.003	-0.026	0.814	-0.04	0.009	0.705	-0.17	0.31	<0.001*	-0.18	0.21	0.002*
**Consequence**	-0.051	0.097	0.277	0.071	-0.014	0.543	0.33	-0.39	<0.001*	0.27	-0.31	<0.001*
**Control personal & treatment**	-0.076	0.15	0.10	0.046	0.026	0.886	-0.055	-0.21	0.308	0.10	-0.052	0.277
**Illness coherence**	-0.061	0.14	0.114	-0.027	0.075	0.421	-0.004	-0.049	0.777	-0.044	0.14	0.153
**Treatment burden**	-0.003	0.006	0.941	-0.102	0.071	0.189	-0.43	0.31	<0.001*	-0.24	0.32	<0.001*
**Prioritization**	0.076	-0.091	0.192	-0.085	0.051	0.301	-0.16	0.27	0.005*	-0.19	0.24	<0.001*
**Causal relation**	-0.10	0.15	0.049*	0.111	-0.044	0.221	0.060	-0.27	0.015*	0.15	-0.16	0.013*
**Activity restrict**	0.031	-0.022	0.699	-0.18	0.13	0.021*	-0.47	0.22	<0.001*	-0.19	0.26	<0.001*
**Emotional representation**	0.021	-0.017	0.779	-0.097	0.070	0.226	-0.38	0.22	0.0004*	-0.23	0.30	<0.001*
**Overall Factor Score**	-0.014	0.038	0.309	-0.018	0.032	0.336	-0.086	0.002	0.168	-0.031	0.066	0.058

* Significant at *α* = 0.05 (t-test was used to test for mean differences)

The correlation between the IPQ-RDE constructs and psychosocial variables (Table 6) indicated the following: For depression, significantly positive correlations were found for timeline, treatment burden, activity restriction, emotional representation, and overall IPQ-RDE score indicating that inaccurate perception (higher scores) was related to greater depression; For social support, significantly negative correlations were found for prioritization, activity restriction, and emotional representation indicating that inaccurate perception was related to lower social support, while higher scores on identity and consequences were related to significantly greater social support. Table 6 (predictive validity) indicates that IPQ-RDE constructs (timeline, treatment burden, prioritization, activity restriction, emotional representation), and overall scores were significantly negatively correlated with oral health quality of life indicating that inaccurate perception (higher scores) were related to lower quality of life.

**Table 6:** Correlation between illness perception constructs (10 factor model)and participant psychosocial and quality of life (QOL) variables.

	Depression	Social Support	QOL
**n**	191	191	191
**Identity**	-0.14	0.14*	0.25*
**Timeline**	0.15*	-0.12	-0.20*
**Consequence**	-0.18*	0.21*	0.28
**Control**	0.10	0.054	0.016
**Illness coherence**	0.14	-0.005	-0.074
**Treatment burden**	0.17*	-0.11	-0.32*
**Prioritization**	0.14	-0.19*	-0.21*
**Causal relation**	0.07	0.14	0.076
**Activity restrict**	0.18*	-0.18*	-0.26*
**Emotional representation**	0.27*	-0.15*	-0.34*
**Overall Factor Score**	0.22*	-0.041	-0.22*

* Significant at *α* = 0.05 (Z-test based on the Spearman correlation was used to test for non-zero correlations)

The DIF analysis did not exhibit differences (p > 0.05) between participant age and housing categories (Table 7) indicating that IPQ-RDE scores can be validly compared between these socio-demographic sub-groups. For race, most of the constructs did not exhibit differences, however, two constructs did (timeline and treatment burden). To note is that we were not able to carry out the DIF analysis for some constructs (or construct-group combinations) due to numerical problems preventing the fit of the full model (with separate parameters for each group), occurring for example when the groups do not have responses for the same categories for an item.

**Table 7:** Differential item functioning (DIF) of illness perception constructs with age, housing, race among older adults.

	Age			Housing			Race		
	N (<75 yrs 112/ >75 yrs 75)			N (Hud 95/non-hud 96)			N (Black 86/ non-black 102)		
	chi square	df	P-value	chi square	df	P-value	chi square	df	P-value
**Consequence**	6.61	6	0.358	9.62	6	0.142	6.7	6	0.348
**Control**	7.70	3	0.261	3.99	3	0.679	21.34	3	0.002
**Timeline**	5.91	3	0.315	5.29	3	0.381	16.38	3	0.006
**Treatment burden**	3.31	5	0.652	8.45	5	0.133	4.76	5	0.446
**Prioritization**	1.80	3	0.615	1.37	3	0.713	0.70	3	0.872
**Causal relationship**	1.04	3	0.793	3.57	3	0.312	-	3	-*
**Activity restriction**	3.34	3	0.329	1.42	3	0.701	6.20	3	0.102

p > .05 indicates lack of evidence for a difference in IPQ-RD item factor loadings between the compared groups (by age or race or housing group)

* Value was not available due to residual covariance matrix is not positively definite

### Reliability

Internal consistency (Cronbach’s alpha) for IPQ-RDE factors were as follows: Identity (0.81), Timeline (0.53), Consequences (0.84), Control (0.88), Illness coherence (0.73), Treatment burden (0.77), Prioritization (0.75), Causal relationship (0.46), Activity restriction (0.84), Emotional representations (0.79).

## Discussion

### Construct validity

To our knowledge, this is the first study to use the Common-Sense Model of Self-Regulation (CSM) to develop an integrated Illness Perception Questionnaire Revised for Dental Use in Older/Elder Adults (IPQ-RDE) with single and multiple oral health conditions. Our results indicate that this integrated measure has good overall validity and reliability for use among community-dwelling adults aged 62 years or older. The confirmatory factor analysis (CFA) validated a 10-factor structure (40 items) aligning with the theoretical constructs of the CSM framework. After related constructs were combined (i.e. Timeline and Control) and three redundant items removed, factor loadings significantly correlated with the ten constructs confirming a good model-data fit (RMSEA < 0.08). Thus, revisions to the initial 43-item version of the IPQ-RDE with appropriate enhancements substantially improved the model fit. Specifically, we used both cognitive interviewing and quantitative data during the development phase and modified the IPQ-RDE items using plain language and a reading level (Flesch-Kinkaid: 7^th^ grade) that was applicable to the target population. Finally, all CSM factors formed unidimensional scales, an important finding since it validates the calculation of summative factor scores. More recently [32, 33], a revised Illness Perception Questionnaire for Oral Health (IPQ-R-OH) has been developed in Spanish for adults 18 years or older with caries and periodontal disease. The IPQ-R-OH was reported to have acceptable construct validity and reliability, however, this is not an integrated measure specific for older adults.

### Concurrent, discriminant, predictive validity

*For concurrent validity*, edentulous status was significantly related to five of the ten IPQ-RDE constructs, while untreated coronal and root decay were not. Participants with periodontitis had a better perception of only the identity construct indicating that they were more aware of the symptoms of the disease compared to those without periodontitis. Participants without teeth however had a poorer understanding of the consequences, control, and causal relationship of oral conditions, and therefore restructuring disease perception may have long lasting influence on retaining teeth than on proximal outcomes (untreated decay). We also found that edentulous participants had an accurate understanding of the timeline and prioritization suggesting that such an understanding may have been a result of their experiences with the oral conditions. Illness perception is formed by cultural knowledge of the illness, information from doctors/dentists and/or others, and illness threat from past and current experiences with the disease [22].

*For discriminant validity*, participants with poor tooth/gum condition had significantly inaccurate understanding of timeline, treatment burden, prioritization, activity restriction, and emotional representation, but an accurate understanding of the identity, consequences, and causal relationship. These findings indicate that edentulous participants had perceptual differences compared to those with poor perceived tooth/gum condition in some of the same constructs (i.e. consequences, causal relationship). So, restructuring beliefs about oral conditions from a younger age is necessary. Some of the constructs of the IPQ-RDE had significant correlation with the depression and social support scale validating our hypothesis regarding external construct validity. The IPQ-RDE also showed excellent predictive validity in that six out of ten constructs were significantly related to oral health quality of life (OHQOL), indicating that inaccurate perception of oral conditions can be predictive of OHQOL. The IPQ-RDE could be used as a prediction tool in future studies.

### Item response theory (IRT)

The DIF analysis indicated that the IPQ-RDE performed uniformly across age and housing subgroups making it potentially applicable for use with older adults aged 62 years or older and living in HUD and non-HUD housing. Some differences were found for race; however, this was for only two of the constructs (timeline and timeline burden), suggesting that the IPQ-RDE overall is applicable across racial groups. Lastly, the reading level is suitable for low-income, low health literacy populations.

### Uniqueness of the new IPQ-RDE measure

The IPQ-RDE is unique for the following reasons: **1.** Uses the CSM, a dynamic perceptual, behavioral, and cognitive framework [34] to assess older adult’s perception/representation of the chronicity of oral diseases. This comprehensive measure is multi-dimensional and fundamentally different from the existing oral health measures that mainly assess the consequences of oral diseases in older adults [15–17]. Often illness perception has been linked to health literacy, but it cannot be achieved without moving the older adult from a disorganized to an organized representation of their oral disease [18]. Many older adults accept their oral health problems as an inevitable process of aging, and our results show that those with complete tooth loss had inaccurate perception; **2.** For the first time, a new integrated measure has been developed for older adults with single and multiple oral diseases. In our sample, about 84% had a single oral condition, and 92% with one or more medical conditions. But in reality, almost all older adults would have had some tooth problems in their lifetime and illness perception may have already been formed from past dental illness. Thus, our results has shown that the IPQ-RDE can work for older adults in various situations. An integrated measure has advantages such as the use of a single instrument rather than administration of a separate illness perception questionnaire for each condition plus the use of the MULTIPLeS as is now being used for medical conditions [35]. Except for identity (i.e. symptoms of disease) and consequences (some items), the items in other constructs have generic wording and mainly assesses the participant’s perception of the chronicity of their oral or systemic condition. Further, for the identity construct we did not ask for specific symptoms of the participant’s oral disease, but used two items to capture two core issues–that symptoms can be intense and that the oral condition has many rather than specific symptoms. This approach, similar to our previous work [18], was possible for oral diseases since they share common identity symptoms (pain, sensitivity, abscess etc.) and consequences (chewing, eating, smiling etc.). It is possible an integrated measure can also be developed for medical conditions that share common symptoms and could have value for an older adult population to self-manage and take action in a timely manner.

### Application of CSM theory to oral diseases

An older adult with untreated caries and/or periodontal disease who also has single/multi-morbid systemic conditions will analyze and interpret the meaning of their health threat using a cognitive process of appraisal and then initiate a coping and action plan to self-manage this threat. For example, the older adult may interpret that they have no symptoms (identity), that oral problems are not serious (consequences), be unaware that oral bacteria may be present (cause), not think that treatment would help (control), and believe that oral problems and eventual tooth loss are a natural process of aging (timeline). Further, multi-morbidity can influence beliefs in the following ways: having more than one oral condition can make the treatment ineffective (treatment burden), may consider one condition more serious than the other (prioritization), may not know that oral conditions can be linked to other oral and medical conditions (causal relationship), may think that managing oral condition limits their daily activities (activity restriction), and getting sad or upset about their oral health (emotional representation). Such an illness representation may lead to an older adult’s lack of concrete actions to self-manage their condition in terms of behavioral changes or seeking dental care. The IPQ-RDE has utility such that it can indicate the constructs that need intervention to restructure disease perception.

The limitations of the study is that it did not include a more diverse groups of older adults who speak other languages or who do not live in single family housing or in community housing facilities in rural areas. Therefore, we recommend that the IPQ-RDE be tested in other population subgroups (e.g. Hispanics) and in adults living in other types of housing and rural areas. Also, the administration of the questionnaire took an average of 17.4 ± 9.7 minutes. For some older adults the length of the questionnaire may be inappropriate. Thus, development of a brief IPQ-RDE that is less time consuming may benefit older adults for use especially in clinical settings.

## Conclusions

In conclusion, our study describes the psychometric properties of the new IPQ-RDE measure (for participants ≥ 62 years) including information on item response theory models, model-data fit, scale dimensionality, and comparability of IPQ-RDE scores across participant age and housing subgroups. This new IPQ-RDE measure could serve as a tool for dental and medical professionals to aid in understanding older adult’s representation of oral diseases and its connection to systemic diseases, in order to promote self-management strategies in seeking dental care and the importance of retaining teeth throughout life. For example, the IPQ-RDE could be used as an outcome measure to assess changes before and after behavioral interventions that restructure disease beliefs or as a mediator in causal pathway analysis. Similarly, dental/medical professionals could use the IPQ-RDE as a clinical tool to evaluate oral disease perception and restructure patient’s beliefs to improve adoption of preventive behaviors.

## Supporting information

S1 Text Illness perception questionnaire—revised for dental use in older adults. (DOCX)

S1 Table Rasch analysis of IPQ-RDE among older adults (10 factors). (DOCX)

S2 Table Differential item functioning (DIF) of illness perception constructs with age, housing, race among older adults using the rasch model. (DOCX)

S1 Dataset Data&codebook for rnrolled participants (N = 198). (XLSX)
