# Supplementary material for: The psychometric properties of a new oral health illness perception measure for adults aged 62 years and older
Source: PLoS One. 2019 Apr 10;14(4):e0214082. doi: 10.1371/journal.pone.0214082 (PMC6457485; doi:10.1371/journal.pone.0214082)
Supplement: S1 Text — (DOCX) [file pone.0214082.s001.docx]

**S1 Text. Illness Perception Questionnaire - Revised for Dental Use in Older Adults**

**ID#: _____________ Date: ____________**

**Indicate whether you strongly agree, agree, neither agree nor disagree, disagree, or strongly disagree with the following statements about your oral health condition.**

| **No.** | **Views about my oral health condition:** | **Strongly Agree** | **Agree** | **Neither Agree nor Disagree** | **Disagree** | **Strongly Disagree** |
| --- | --- | --- | --- | --- | --- | --- |
| **Identity** | | | | | | |
| 1 | My oral health condition is an illness with symptoms generally of an intense nature. | 1 | 2 | 3 | 4 | 5 |
| 2 | My oral health condition is an illness with many symptoms. | 1 | 2 | 3 | 4 | 5 |
| **Timeline** | | | | | | |
| 3 | My oral health condition will last a short time. | 1 | 2 | 3 | 4 | 5 |
| 4 | I expect to have an oral health condition for the rest of my life. | 1 | 2 | 3 | 4 | 5 |
| 5 | My oral health condition will improve over time. | 1 | 2 | 3 | 4 | 5 |
| **Consequence** | | | | | | |
| 6 | My oral health condition is a serious problem. | 1 | 2 | 3 | 4 | 5 |
| 7 | My oral health condition is as serious as any other medical condition. | 1 | 2 | 3 | 4 | 5 |
| 8 | My oral health condition has major consequences on my life such as chewing, speaking or aesthetic problems. | 1 | 2 | 3 | 4 | 5 |
| 9 | My oral health condition has much effect on my daily life. | 1 | 2 | 3 | 4 | 5 |
| 10 | My oral health condition has a big effect on how others think about me. | 1 | 2 | 3 | 4 | 5 |
| 11 | My oral health condition has caused money problems for me or my family. | 1 | 2 | 3 | 4 | 5 |
| **Personal Control** | | | | | | |
| 12 | There is a lot I can do to control my symptoms. | 1 | 2 | 3 | 4 | 5 |
| 13 | What I do decides if my oral health condition gets better or worse. | 1 | 2 | 3 | 4 | 5 |
| 14 | I have the power to influence the outcome of my oral health condition. | 1 | 2 | 3 | 4 | 5 |
| **Treatment Control** | | | | | | |
| 15 | There is a lot that can be done to improve my oral health condition. | 1 | 2 | 3 | 4 | 5 |
| 16 | My treatment will help make my oral health condition better. | 1 | 2 | 3 | 4 | 5 |
| 17 | My treatment can control my oral health condition. | 1 | 2 | 3 | 4 | 5 |
| **No.** | **Views about my oral health condition:** | **Strongly Agree** | **Agree** | **Neither Agree nor Disagree** | **Disagree** | **Strongly Disagree** |
| **Illness Coherence** | | | | | | |
| 18 | The symptoms of my oral health condition are puzzling to me. | 1 | 2 | 3 | 4 | 5 |
| 19 | My oral health condition makes sense to me. | 1 | 2 | 3 | 4 | 5 |
| 20 | I have a clear picture or understanding of my oral health condition. | 1 | 2 | 3 | 4 | 5 |
| **Timeline Cycle** | | | | | | |
| 21 | The symptoms of my oral health condition may change from day to day. | 1 | 2 | 3 | 4 | 5 |
| 22 | I cannot predict how my oral health condition will change over time. | 1 | 2 | 3 | 4 | 5 |
| 23 | I go through cycles in which my oral health condition gets better and worse. | 1 | 2 | 3 | 4 | 5 |
| **Treatment Burden** | | | | | | |
| 24 | I feel overwhelmed by the treatment for my oral health condition. | 1 | 2 | 3 | 4 | 5 |
| 25 | It is difficult to visit a dentist for my oral health condition when I have a problem. | 1 | 2 | 3 | 4 | 5 |
|  | ***For questions 26-31, if you had one oral health condition imagine you have more than one oral health condition.*** |  |  |  |  |  |
| 26 | Visiting a dentist for each of my oral health conditions would cause more problems. | 1 | 2 | 3 | 4 | 5 |
| 27 | Having more than one oral health condition would make treatments less effective. | 1 | 2 | 3 | 4 | 5 |
| 28 | Having more than one oral health condition would make it difficult to get the best available treatment. | 1 | 2 | 3 | 4 | 5 |
| **Prioritization** | | | | | | |
| 29 | With an oral health condition, one is more serious than the others. | 1 | 2 | 3 | 4 | 5 |
| 30 | With an oral health condition, one takes over the others. | 1 | 2 | 3 | 4 | 5 |
| 31 | With an oral health condition, one has more of an effect on my life than the others. | 1 | 2 | 3 | 4 | 5 |
| **Causal Relationship** | | | | | | |
| 32 | The causes of oral health conditions are linked. | 1 | 2 | 3 | 4 | 5 |
| 33 | One oral health condition causes another. | 1 | 2 | 3 | 4 | 5 |
| 34 | An oral health condition is a side effect of medication used for a medical condition. | 1 | 2 | 3 | 4 | 5 |
| 35 | My oral health condition can be linked to a medical condition. | 1 | 2 | 3 | 4 | 5 |
| \| **No.** \| **Views about my oral health condition:** \| **Strongly Agree** \| **Agree** \| **Neither Agree nor Disagree** \| **Disagree** \| **Strongly Disagree** \| \| --- \| --- \| --- \| --- \| --- \| --- \| --- \| | | | | | | |
| **Activity Restriction** | | | | | | |
| 36 | Time spent managing my oral health condition makes it difficult to do my daily activities. | 1 | 2 | 3 | 4 | 5 |
| 37 | Time spent managing my oral health condition has limited my activities. | 1 | 2 | 3 | 4 | 5 |
| 38 | Time spent managing my oral health condition has reduced my social life. | 1 | 2 | 3 | 4 | 5 |
| **Emotional Representations** | | | | | | |
| 39 | I get really sad and upset when I think about my oral health condition. | 1 | 2 | 3 | 4 | 5 |
| 40 | My oral health condition makes me feel angry. | 1 | 2 | 3 | 4 | 5 |
| 41 | My oral health condition worries me. | 1 | 2 | 3 | 4 | 5 |
| 42 | Having more than one oral health condition makes someone more bad-tempered. | 1 | 2 | 3 | 4 | 5 |
| 43 | When I feel sad or down, managing my oral health condition is hard to do. | 1 | 2 | 3 | 4 | 5 |

**Indicate whether you strongly agree, agree, neither agree nor disagree, disagree or strongly disagree with the following statements about the possible causes of your oral health condition.**

| **No.** | **Possible Causes:** | **Strongly Agree** | **Agree** | **Neither Agree nor Disagree** | **Disagree** | **Strongly Disagree** |
| --- | --- | --- | --- | --- | --- | --- |
| 1 | Stress or worry | 1 | 2 | 3 | 4 | 5 |
| 2 | Hereditary (e.g. it runs in the family) | 1 | 2 | 3 | 4 | 5 |
| 3 | A germ or virus | 1 | 2 | 3 | 4 | 5 |
| 4 | Diet or eating habits | 1 | 2 | 3 | 4 | 5 |
| 5 | Chance or bad luck | 1 | 2 | 3 | 4 | 5 |
| 6 | Poor dental care in my past | 1 | 2 | 3 | 4 | 5 |
| 7 | My own behavior | 1 | 2 | 3 | 4 | 5 |
| 8 | Mental attitude (e.g. thinking about life negatively) | 1 | 2 | 3 | 4 | 5 |
| 9 | My emotional state (e.g. feeling down, lonely, anxious) | 1 | 2 | 3 | 4 | 5 |
| 10 | Getting older | 1 | 2 | 3 | 4 | 5 |
| 11 | Alcohol | 1 | 2 | 3 | 4 | 5 |
| 12 | Smoking | 1 | 2 | 3 | 4 | 5 |

**Name in order the three most important factors that you believe caused your oral health condition. Use any of the items from the previous list or name additional factors of your own.**

1. ___________________________________________

2. ___________________________________________

3. ___________________________________________
