# Supplementary material for: The psychometric properties of a new oral health illness perception measure for adults aged 62 years and older
Source: PLoS One. 2019 Apr 10;14(4):e0214082. doi: 10.1371/journal.pone.0214082 (PMC6457485; doi:10.1371/journal.pone.0214082)
Supplement: S2 Table — (DOCX) [file pone.0214082.s003.docx]

**S2 Table. Differential Item Functioning (DIF) of Illness Perception Constructs with Age, Housing, Race Among Older Adults Using the Rasch Model**

|  | **Age** | | | **Housing** | | | **Race** | | |
| --- | --- | --- | --- | --- | --- | --- | --- | --- | --- |
|  | **N (<=75 yrs / >75 yrs)** | | | **N (Hud /non-hud)** | | | **N (Black / non-black)** | | |
|  | chi square | df | P-value | chi square | df | P-value | chi square | df | P-value |
| **Identity** | 11.18 | 10 | 0.344 | 5.36 | 10 | 0.866 | 17.88 | 10 | 0.057 |
| **Timeline** | - | 25 | -* | 21.50 | 25 | 0.664 | 34.68 | 25 | 0.094 |
| **Consequence** | 45.10 | 30 | 0.038 | 35.18 | 30 | 0.236 | 56.90 | 30 | 0.002 |
| **Control** | 23.00 | 30 | 0.815 | 22.98 | 30 | 0.816 | - | 30 | -* |
| **Illness Coherence** | 2.96 | 10 | 0.982 | 7.48 | 10 | 0.679 | - | 10 | -* |
| **Treatment burden** | - | 25 | -* | - | 25 |  | - | 25 | -* |
| **Prioritization** | 13.06 | 15 | 0.598 | - | 15 |  | - | 15 | -* |
| **Causal relationship** | - | 15 | -* | 18.94 | 15 | 0.216 | - | 15 | -* |
| **Activity restriction** | - | 15 | -* | - | 15 |  | - | 15 | -* |
| **Emotional Representations** | - | 25 | -* | 21.88 | 25 | 0.643 | 44.94 | 25 | 0.008 |

p > .05 indicates lack of evidence for a difference in IPQ-RD item factor loadings between the compared groups (by age or race or housing group).

* Value was not available due to residual covariance matrix not being positively definite.
